# The protective roles of NLRP6 in intestinal epithelial cells

**DOI:** 10.1111/cpr.12555

**Published:** 2018-12-04

**Authors:** Jiuheng Yin, Baifa Sheng, Kunqiu Yang, Lihua Sun, Weidong Xiao, Hua Yang

**Affiliations:** ^1^ Department of General Surgery Xinqiao Hospital Third Military Medical University Chongqing China; ^2^ Department of General Surgery Navy General Hospital Beijing China

**Keywords:** antimicrobial peptides, goblet cell, IL‐18, microbiota, mucus, NLRP6

## Abstract

The evolution of chronic inflammatory diseases is thought to be due to a combination of host genetic variations and environmental factors that include the alteration of intestinal flora, termed “dysbiosis.” The intestinal mucosal barrier includes a chemical barrier and physical barrier that have important roles in protecting the intestine against inflammatory injury. The chemical barrier includes antimicrobial peptides (AMPs), and the physical barrier includes a mucous layer, a monolayer of intestinal epithelial cells and cell junctions. The intestinal mucosal barrier is not a static barrier, but rather, it strongly interacts with the gut microbiome and cells of the immune system. Correct expression of AMPs, together with mucus and balanced epithelial cell proliferation, prevents the occurrence of disease. NLRP6, a member of the nucleotide‐binding domain, leucine‐rich repeat‐containing (NLR) innate immune receptor family, participates in the progression of intestinal inflammation and enteric pathogen infections. It has become apparent in recent years that NLRP6 is important in disease pathogenesis, as it responds to internal ligands that lead to the release of AMPs and mucus, thus regulating the regeneration of intestinal epithelial cells. This review summarizes the activation of NLRP6 and its protective role in the intestinal epithelial cell.

## 1 INTRODUCTION

In the gut, there are approximately 10‐100 trillion bacteria that are essential for health.^1^, ^2^ When pathogenic microorganisms break through the intestinal mucosal barriers and invade parenchymal tissues, the result is pathological inflammatory consequences including intestinal inflammation, coeliac disease and enteric pathogen infections.^3^, ^4^ To prevent these disorders, the intestinal mucosal barrier uses tightly regulated mechanisms to control the integrity of the intestinal barrier, including a mucous layer, epithelial cells, tight/adherens junctions and desmosomes.^5^, ^6^, ^7^ The layer of mucus that is secreted by specialized epithelial cells called goblet cells (GCs) distributes on the surface of epithelium.^8^ There are large numbers of AMPs in the mucous layer that limit the amounts of gut bacteria and pathogens that can access host cells.^9^, ^10^ The intestinal epithelial cell (IEC) layer is a continuous single layer whose cells are sealed together by tight junction (TJ) proteins, tight junctions, adherens junctions and desmosomes.^7^ Proper homeostasis and renewal of epithelial cells play a key role in preventing the formation and development of inflammatory bowel disease.^11^, ^12^

Inflammasome complexes are assembled by activation of nucleotide‐binding domain, leucine‐rich repeat‐containing proteins (NLRs), AIM2‐like receptors (ALRs), or pyrin.^13^ Members of the NOD‐like receptor (NLR) family play fundamental roles in immunity and host defence, and, when dysregulated, contribute to intestinal disease pathogenesis. NLRs are composed of 23 family members in the human genome and 34 in the mouse genome.^14^ In recent years, Ranson et al^15^ observed that the inflammasome component NLRP6 is up‐regulated in human ileal Crohn's disease(CD), but not to a significant extent in human ulcerative colitis. In contrast, Misagh Alipour et al^16^ found a trend for reduced NLRP6 expression in IBD subsets but this did not reach statistical significance. Further study indicated that the expression of NLRP6 was reduced in the epithelial layer of CD and ulcerative colitis (UC) patients. Animal models further confound the association between NLRP6 expression and the integrity of the intestinal barrier. In particular, decreased levels of NLRP6 have been reported in small intestinal inflammation (enteritis) induced by WAS (water‐avoidance stress) in mice.^17^ However, the expression of NLRP6 is unclear in the mouse model of colitis. Therefore, the precise regulation of NLRP6 expression varies according to biological context (eg, under normal homeostatic vs during pathologic chronic inflammation), region of the gastrointestinal tract, and, potentially, according to species. Future studies are needed to fully define these differences. To help guide these efforts, here, we review the most current literature on NLRP6 to summarize what is known about its role in maintaining the integrity of the intestinal mucosal barrier.

## 2 NLRP6 SIGNALLING PATHWAYS DURING ACTIVATION

NLRP6, a member of the NLR innate immune receptor family, plays an important role in attenuating inflammatory signals after a pathogen has been eliminated to prevent the onset of chronic inflammation. When NLRP6 is activated by various factors, the adaptor protein known as apoptosis‐associated speck‐like protein containing CARD (ASC) is recruited, followed by functional inflammasome formation.^18^ Subsequently, the functional inflammasome leads to proteolytic cleavage and activation of caspase‐1/11, followed by release of the proinflammatory cytokines interleukin‐(IL)‐1b and IL‐18.^19^ In addition to classical activation, the noncanonical NLRP6 inflammasome also promotes pyroptotic cell death and the release of cytokines activated by procaspase‐1/11 that belong to the aspartate‐specific cysteine protease family.^20^ In recent years, Radulovic et al^21^ found that NLRP6 signalling could be independent of the activation of the inflammasome and played an important role in protecting mice against colitis by reprogramming the gut microbiota. They reported that Flavones alleviated inflammatory relied on the regulation of the gut microbiota by the NLRP6. In contrast, the symptoms of colitis were alleviated upon apigenin administration even in the absence of either caspase‐1/11 or ASC.

## 3 THE LIGAND OF NLRP6 IN IECS

The highly expressed NLRP6 in mouse IEC plays an important role in protecting against the development of enteritis by regulating the stability of the intestinal mucosal barrier.^17^, ^22^, ^23^, ^24^ Therefore, clarifying the factors regulating NLRP6 or identifying the ligands for NLRP6 could shed light on adaptation of the intestine when exposed to microbes. Sun et al^17^ reported that water‐avoidance stress (WAS) inhibited NLRP6 expression and WAS‐induced disruption of NLRP6 inflammasome signalling led to pathological changes of small intestine in the model of stress‐induced enteritis in mice. As a mediator of the stress effects on the intestinal epithelium, corticotropin‐releasing hormone (CRH) mediated the WAS‐induced pathological changes of intestine. Direct treatment of colonic epithelial cell with CRH inhibited the expression and function of NLRP6.^25^, ^26^ Just as NLRP6 regulated the gut flora, WAS also induced gut dysbiosis and transmissible enteritis. In a microarray analysis, Kempster et al^27^ observed a potential PPARG‐RXRA binding site containing a NLRP6 gene binding site. Furthermore, the PPAR‐γ agonist rosiglitazone significantly increased the expression of NLRP6 mRNA in Caco2 cells.

Although rosiglitazone stimulated expression of NLRP6, the ligand of NLRP6 remains unknown.^27^ A recent report aimed to identify the primary NLRP6 ligand in the metabolic products of gut bacteria.^23^ The cross‐talk between commensal bacteria and their host during homeostasis and dysbiosis holds the key to understanding many idiopathic diseases.^28^ NLRP6 regulates the composition of intestinal flora, and metabolites are considered pivotal mediators of host‐microbiota communication.^17^, ^23^, ^28^, ^29^ Therefore, microbiota‐modulated metabolites may take part in regulating NLRP6 inflammasome signalling and downstream antimicrobial pathways. Through a metabolomic screen of caecal contents, researchers found that taurine induced secretion of epithelium‐derived Il‐18 and upregulation of AMPs in a dose‐dependent manner.^23^ Administering taurine to mice in drinking water ameliorated levels of histamine and spermine that were involved in the microbiota‐induced suppression of inflammasome signalling.

Cross‐talk between the intestinal epithelium and the microbiota maintains the microenvironment and prevents chronic inflammation. This communication is partly mediated through the recognition of bacterial proteins by host‐encoded innate receptors, including Toll‐like receptors (TLRs).^30^, ^31^, ^32^ TLRs recognize LPS, the triacylated lipopeptide P3CSK4 and flagellin, after which reactive oxygen species (ROS) synthesis and MyD88 signalling takes place up‐stream of inflammasome activation.^33^ The flavone apigenin, found in plant‐derived food sources, also has been shown that have anti‐inflammatory and anti‐proliferative effects that relied on regulation of gut microbiota via NLRP6.^21^

## 4 THE NLRP6‐IL‐18 AXIS AND THE MICROBIOME IN IECS

Functional NLRP6 inflammasomes lead to proteolytic cleavage and activation of caspase‐1, after which the proinflammatory cytokine IL‐18 is released.^18^ To assess the possibility that NLRP6 might have an influence on colitis, NLRP6^−/−^ mice were used in several studies.^14^, ^34^, ^35^, ^36^ In this model, NLRP6‐deficient mice displayed enhanced susceptibility to DSS‐induced colitis. The researchers also observed that levels of IL‐18 were reduced. Furthermore, 16S rRNA analysis of the flora composition revealed a more colitogenic microbiota in the intestine of NLRP6‐deficient mice.

We learned that activation of inflammasomes resulted in multiple downstream effects, including production of active forms of pro‐IL‐18 by proteolytic cleavage.^37^ Levy et al. found that knocking out IL‐18 resulted in significant exacerbation of colitis severity and communities of intestinal bacteria similar to those of NLRP6‐deficient mice. These results indicated that the influence of NLRP6 on intestinal bacterial communities might partly depend on IL‐18 and IL‐18 had a protective role in DSS‐induced colitis. Interestingly, in a rat model of neonatal necrotizing enterocolitis (NEC) (a life‐threatening complication associated with preterm birth 38), the expression of IL‐18 increased and the degree of inflammation correlated with IL‐18 gene expression.^39^, ^40^ Furthermore, Halpern et al. reported that knocking out IL‐18 reduced the incidence and severity of NEC. Kempster et al^27^ ascribed the difference to the excessive activation of IL‐18 in the morbid state. However, the different expression of IL‐18 might also contribute to “conflicting” observations regarding the role of IL‐18 in NEC (occurring in the ileum) as opposed to its role in DSS‐induced colitis (most severe in the distal colon). We believe that further studies regarding the relationship between the expression location and function of NLRP6 should be carried out in the future. To this end, the deficiency of NLRP6 in mouse colonic epithelial cells altered faecal microbiota that was characterized by expanded representation of the bacterial phylum Bacteroidetes (Prevotellaceae) and TM7.^34^

In the gut, AMPs are crucial components of the innate immune system that participate in the organization of the “healthy” host‐microbial interface with mechanisms ranging from IgA and mucus secretion to AMP production.^41^, ^42^ Studies revealed that NLRP6 not only regulated the transcription of the AMPs, including angiogenin‐4, intelectin‐1 and resistin‐like beta, via IL‐18 in an autocrine manner on the epithelium 23 but also downregulated the expression of IL‐22 binding protein (IL‐22BP) indirectly. As a result of downregulated IL‐22BP, there were increased levels of bioactive IL‐22, a potential inducer of the epithelial antimicrobial response.^43^

## 5 THE NLRP6‐MUC2 AXIS AND MICROBIOME IN COLONIC EPITHELIAL CELLS

The thick mucous layer is a key component that defends the lamina propria against being penetrated by microbes and pathogens.^44^ In UC patients and in mice with DSS‐induced colitis, there was often depletion of recognizable goblet cells, decreased mucus synthesis and secretion in the colonic epithelium.^45^, ^46^ In an enteric pathogen clearance experiment, Wlodarska et al^29^ observed increased *C. rodentium* burden and pathological changes in NLRP6‐deficient mice compared with those of WT mice. Asc^−/−^ and caspase‐1/11^−/−^ mice also showed inability to clear *C. rodentium*. These results suggest that NLRP6 inflammasome activation is pivotal for host defence against A/E pathogen infection.

Muc2 mucin is a large glycoprotein produced by goblet cells that forms the first line of innate host defence.^47^ Muc2‐deficient (Muc2^−/−^) mice develop spontaneous colitis and cannot prevent attachment and removal of adherent pathogens from the mucosal surface.^48^ After inoculating Muc2^−/−^ mice with *C. rodentium*, a murine A/E pathogen related to diarrhoeagenic A/*E. coli*, there was 10‐100‐fold greater *C. rodentium* burden in Muc2^−/−^ than in WT mice.^49^ NLRP6 is highly expressed throughout the intestinal mucosa, specifically in goblet cells. This finding suggests that NLRP6 regulates mucus secretion. Further studies revealed that the intestinal epithelium of NLPR6^−/−^ mice exhibited remarkable hyperplasia of goblet cells. However, goblet cell mucus granule secretion was inhibited, and a thick continuous overlaying inner mucous layer was lacking.^29^

The primary goblet cell defect in the absence of NLPR6 is independent of IL‐1 and IL‐18. This suggests that the mechanism of regulating mucous secretion is different from the mechanism by which NLRP6 regulates AMPs. Interestingly, NLRP6 only regulates the fusion of mucus granules and intestinal epithelium and subsequent mucus secretion but does not influence the expression of mucus. Previous studies reported that autophagy was critical for proper function of secretory pathways in Paneth cells, osteoclasts and mast cells.^50^, ^51^, ^52^ Likewise, the lack of visible autophagosome formation in NLRP6‐deficient epithelium indicated that the autophagic processes were required for proper secretion of mucus granules that was mediated by NLRP6. Mucous secretion is dependent on intracellular calcium concentration; therefore, further studies should be carried out to observe whether NLRP6 or autophagy regulates calcium concentrations. Moreover, goblet cells in caspase‐1/11^−/−^ and Asc^−/−^ mice showed a marked lack of mucous secretion.

Colonic GCs express TLRs that recognize LPS, P3CSK4 and flagellin.^53^ Activation of the NLRP6 inflammasome is essential to flagellin response. LPS and P3CSK4 induced Muco2 secretion by TLR‐MyD88 and TLR‐TRIF signalling.^33^ In the gut, these are called sentinel GCs (sen‐GCs) because there is less than one cell per crypt; in other words, these cells are quite rare.^54^ These cells swallow ligands, thereby achieving the activated state. NLRP6 controls sen‐GC expulsion that substantially promotes activated sen‐GCs to trigger an intercellular signal that induces Muc2 secretion in adjacent responsive GCs. Birchenough et al. identified sentinel goblet cells localized to crypt openings.

## 6 NLRP6 AND CELL PROLIFERATION IN INTESTINAL EPITHELIAL CELLS

The intestinal epithelium includes stem cells that ensure tissue homeostasis and regeneration upon tissue damage.^55^ As early as 2011, researchers reported that NLRP6‐deficient mice failed to repair injured epithelium and that expression of IL‐18 decreased during the acute inflammatory response.^24^ How does NLRP6 influence epithelial reconstruction? IL‐22 is well‐known to promote epithelial cell proliferation.^56^ As a soluble IL‐22 receptor, IL‐22BP specifically binds to IL‐22 and prevents the binding of IL‐22 to membrane‐bound IL‐22R1 to inhibit epithelial cell proliferation.^57^ Marta et al found that NLRP6 inflammasomes led to IL‐18‐dependent down‐regulation of IL‐22BP, thereby increasing epithelial cell proliferation. Furthermore, IL‐18 also promoted epithelial cell proliferation by MyD88 signalling.^58^

In mouse models of colitis‐associated cancer induced by treatment with AOM or DSS, NLRP6‐deficient mice developed significantly more and larger tumours than did wild‐type mice.^24^ In that study, the authors addressed the protective role of NLRP6 in tumour development for the first time. Further studies demonstrated that NLRP6 negatively regulated factors involved in epithelial proliferation, including Wnt and Notch target genes in tumours.^59^, ^60^ As the aforementioned studies reported, NLRP6 adjusts the composition of intestinal flora. A colitogenic gut microflora harboured in NLRP6^−/−^ mice and the microbiota‐induced chemokine (C‐C motif) ligand 5 (CCL5) together promoted epithelial cell proliferation through local activation of the IL‐6 pathway.^61^

## 7 OUTLOOK

**Figure 1:**
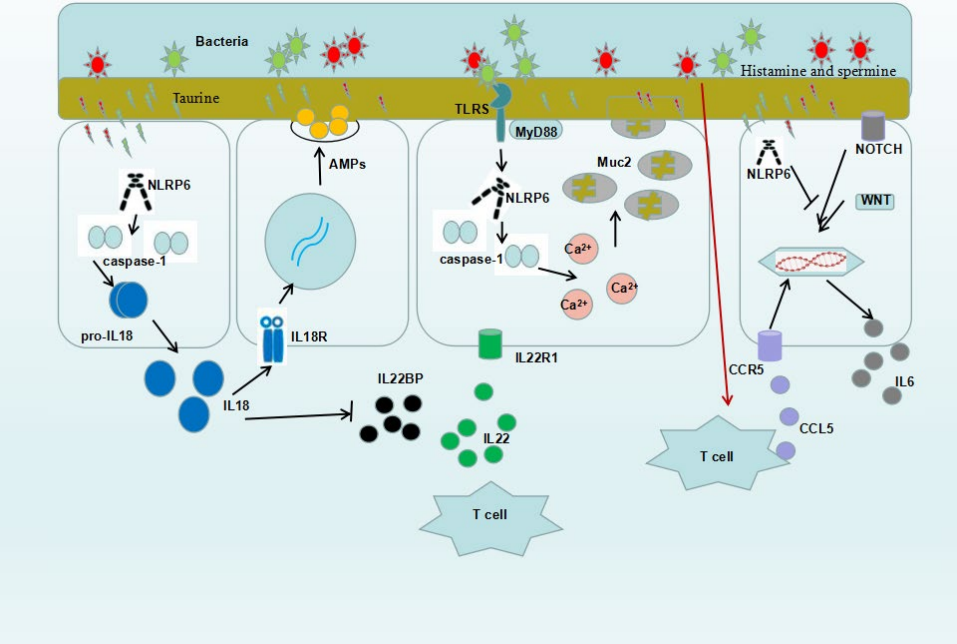
Multiple mechanisms involved in the protective roles of NLRP6 in intestinal epithelial cells. Microbiota‐modulated metabolites activate the NLRP6 inflammasome signalling. Activation of NLRP6 promotes the expression of IL‐18, which stimulate the secretion of AMP to clear the bacteria and inhibit expression of IL‐22BP to enhance the function of IL‐22. NLRP6 takes part in the secretion of mucus meditated by Toll receptor and also directly involved in the secretion of mucus by inhibiting the notch and wnt signalling in goblet cell. NLRP6 indirectly promote epithelial cell proliferation through local activation of the IL‐6 pathway

According to our review, we draw a diagram to easily understand the function of NLRP6 in IEC (Figure 1). The therapeutic potential of inhibiting proinflammatory responses in infectious and autoimmune diseases is raised by successfully using intestinal probiotics to control autoinflammatory diseases associated with aberrant inflammasome signalling. Recent studies have shown that intestinal inflammation is significantly aggravated in the absence of NLRP6. This aggravation often involves decreased Il‐18 and antimicrobial peptide expression, as well as impaired re‐epithelialization and mucus secretion in IEC. NLRP6 also has a protective role in the formation of tumours. Therefore, it is clear that NLRP6 is an important factor in the intestinal injury response, regulating several aspects of healing of inflammation.

Given the differential expression and the specific roles of NLRP6 in intestinal epithelial cells, there is a need for more investigations into the roles of NLRP6 and its ligands in IEC. The majority of studies to date have indicated the activation of NLRP6 and its protective role in intestinal epithelial cells.
